# Counting on commitment; the quality of primary care-led diabetes management in a system with minimal incentives

**DOI:** 10.1186/1472-6963-11-348

**Published:** 2011-12-28

**Authors:** Sheena Mc Hugh, Paul Marsden, Carmel Brennan, Katie Murphy, Celine Croarkin, Joe Moran, Velma Harkins, Ivan J Perry

**Affiliations:** 1 Department of Epidemiology & Public Health, Western Gateway, University College Cork, Cork, Ireland; 2 Department of Public Health, Health Service Executive, Tullamore, Co. Offaly, Ireland; 3 Diabetes Interest Group, Department of General Practice, University College Cork, Cork, Ireland; 4 HSE North East Diabetes Watch, Diabetes/Cardiovascular Facilitator, HSE Primary Care Services, Local Health Office, Rooskey, Co. Monaghan, Ireland; 5 The Health Centre, Banagher, Co. Offaly, Ireland

## Abstract

**Background:**

The aim of the present study was to assess the performance of three primary care-led initiatives providing structured care to patients with Type 2 diabetes in Ireland, a country with minimal incentives to promote the quality of care.

**Methods:**

Data, from three primary care initiatives, were available for 3010 adult patients with Type 2 diabetes. Results were benchmarked against the national guidelines for the management of Type 2 diabetes in the community and results from the National Diabetes Audit (NDA) for England (2008/2009) and the Scottish Diabetes Survey (2009).

**Results:**

The recording of clinical processes of care was similar to results in the UK however the recording of lifestyle factors was markedly lower. Recording of HbA1c, blood pressure and lipids exceeded 85%. Recording of retinopathy screening (71%) was also comparable to England (77%) and Scotland (90%). Only 63% of patients had smoking status recorded compared to 99% in Scotland while 70% had BMI recorded compared to 89% in England. A similar proportion of patients in this initiative and the UK achieved clinical targets. Thirty-five percent of patients achieved a target HbA1c of < 6.5% (< 48 mmol/mol) compared to 25% in England. Applying the NICE target for blood pressure (≤ 140/80 mmHg), 54% of patients reached this target comparable to 60% in England. Slightly less patients were categorised as obese (> 30 kg/m^2^) in Ireland (50%, n = 1060) compared to Scotland (54%).

**Conclusions:**

This study has demonstrated what can be achieved by proactive and interested health professionals in the absence of national infrastructure to support high quality diabetes care. The quality of primary care-led diabetes management in the three initiatives studied appears broadly consistent with results from the UK with the exception of recording lifestyle factors. The challenge facing health systems is to establish quality assurance a responsibility for all health care professionals rather than the subject of special interest for a few.

## Background

The ability of a health system to deliver effective diabetes care reflects a system's wider capacity to manage other chronic diseases [1,2]. The management of chronic conditions such as diabetes requires planned, structured care which is integrated in the wider health system [3]. Enhancing the structure and coordination of care within and between settings has been shown to improve the quality of care for patients with diabetes [4,5]. As a result there is growing emphasis internationally and nationally on system, practice and professional-level initiatives to improve the quality of care.

In Ireland, care is delivered in a variety of ways to patients with diabetes including traditional mixed care, hospital-led care, shared care arrangements and primary care-led management. At general practice level, care is often ad-hoc reflected in the absence of patient registries, irregular review and the lack of guideline use among General Practitioners (GPs) [6]. Access to essential support services such as retinopathy screening is variable and inconsistent with multiple providers of services often influenced by whether a patient is attending the public or private health system.

Within this opportunistic system, there are a number of 'islands of excellence' providing comprehensive systematic care for patients with diabetes. Among these groups are three primary care initiatives involving interested trained professionals aiming to improve the quality of care and patient outcomes. The initiatives seek to develop a multidisciplinary structured approach to diabetes care delivery. Adopting a structured approach to care delivery involves continuing professional education, incorporating guidelines, creating practice registers and ongoing audit and feedback for participating practices. Specialist input is provided in one initiative by a diabetes nurse specialist while the other two initiatives are coordinated by a diabetes nurse facilitator. Participating practices also receive administrative and audit/research support to manage the initiatives.

The aim of this study was to assess the quality of care provided by three primary care-led initiatives adopting a structured approach to Type 2 diabetes care in Ireland, benchmarked against national guidelines and diabetes audit results observed in England and Scotland. The quality of care was assessed in terms of processes of diabetes care and intermediate outcomes of care. Diabetes care delivery in the UK is supported by an infrastructure including a dedicated policy framework, a retinopathy screening programme, robust IT systems in general practice and a financial incentive structure to promote quality assurance. With this in mind we hypothesized that a group of 'champions' with a special interest in diabetes would have a performance comparable to that observed in the UK.

## Methods

### Study design, setting and selection of participants

This cross sectional study was based on data from primary care-based diabetes initiatives across three regions of Ireland; the Diabetes Interest Group Cork (DIG) in the South of Ireland, the HSE Midland Diabetes Structured Care Programme and HSE North East Diabetes Watch. Each initiative provides structured care to patients with diabetes in the general practice setting with some patients also attending secondary care.

### Sample

#### Practices

A purposive sample of three primary care initiatives participated in this study. Three of the most well-established schemes were chosen, all of which conducted and disseminated regular audit. There are currently ten diabetes initiatives in Ireland however most did not engage in routine audit at the time. Although a purposive sample of schemes was used, within each scheme all practices took part in the audit (n = 63). The sample represents nearly 1%of the total number of GPs in Ireland.

Participating practices were from a mixture of urban and rural areas and included single-handed and group practices. All practices provided structured care to patients in the general practice setting which involved continuing professional education, incorporating guidelines, maintaining practices registers and engaging in regular audit and feedback. Each practice employed a Practice Nurse. Specialist input was provided in one initiative by a Diabetes Nurse Specialist while the other two initiatives were coordinated by a Diabetes Nurse Facilitator. Practices also received administrative and audit/research support to manage the initiatives. Two of the three initiatives financially reimbursed practices for their involvement in the scheme.

#### Patients

Adult patients (≥ 18 years of age) with Type 2 diabetes, who were registered with a participating practice on their electronic practice register were eligible for inclusion in the analysis. Type 2 diabetes was defined on the basis of standard clinical and blood glucose criteria [7]. Patients with Type 1 diabetes and Pre-diabetes were excluded from the analysis as the care of these patients is not part of routine audit in all three initiatives.

In the Diabetes Interest Group all patients with Type 2 diabetes registered with participating practices were included in the audit. In the Diabetes Watch programme, all patients who attended the GP for their 2^nd ^diabetes visit were included in the audit. Due to the large number of patients enrolled on the HSE Midland Diabetes Structured Care Programme (> 3000 patients) a random sample was selected from each practice and included in the overall analysis. The sample size was calculated using glycaemic control (HbA1c level) as the outcome measure with a confidence level of 95% and a difference of 2%. In a previous audit, the mean HbA1c for the total sample was 7.6% and the 95% confidence interval was ± 0.111% which equates to ~1.5%. A sample size calculator returned an estimated sample size of 1,168 (51.3% of the total sample). Data were available on 989 patients with Type 2 diabetes (47.1% of total sample) which was 97 patients less than the determined sample size as a number of patients recorded on the database as current and active had died, left the practice or had been transferred to a nursing home.

#### Prevalence

Data were available for 3,010 patients with Type 2 diabetes from 63 practices. It was not possible to calculate the prevalence of Type 2 diabetes in this sample as it is not mandatory in Ireland for patients to register with a single general practice therefore we lack a reliable population denominator. The predicted prevalence for each of the three regions in which the initiatives are based, (4.3% Dublin North Leinster, 4.4% Dublin Mid-Leinster, 4.9% Southern region) was comparable with the estimated national prevalence (4.6%) [8] and the prevalence of Type 2 diabetes in Scotland (4.4%) and England (4.1%).

### Data Collection

The study was based on secondary data analysis of anonymised data which had previously been published and disseminated in individual audit reports from each of the three initiatives therefore ethical approval was not sought for the collated secondary analysis. The data was originally collected for the purpose of service evaluation which did not require patient consent.

Data were collected during 2008/2009 by Diabetes Clinical Nurse Specialists in the Midlands region, by a Diabetes Nurse Facilitator in the South, and in the North East region datasheets were completed and submitted by the practices themselves. Data sources included patient's clinical notes (electronic and paper), letters regarding hospital outpatient appointment and referrals to other services (chiropody/podiatry, retinopathy, dietetics etc). Table 1 shows details of variables collected across all three initiatives.

**Table 1:** Common dataset across three primary care-led diabetes initiatives

Demographics	Process of care	Intermediate Outcome of Care
Gender	Recording of HbA1c	HbA1c

Age	Recording of Blood Pressure	Blood Pressure

	Recording of Body Mass Index	Body Mass Index (BMI)
	
	Recording of Total Cholesterol concentration	Total Cholesterol concentration
	
	Recording of LDL Cholesterol concentration	LDL Cholesterol concentration
	
	Recording of HDL Cholesterol concentration	HDL Cholesterol concentration
	
	Recording of smoking status	Smoking Status
	
	Retinopathy screening in past year	
		
	Foot Assessment in the past year	
		
	Treatment with statin/aspirin	

The national guidelines for Type 2 diabetes care in the community were used to define the optimal standard of care in Ireland [7]. The guidelines outline the appropriate processes of care involved in diabetes management, set targets for the achievement of intermediate outcomes and specify the relevant cut-off points which are included in the text throughout the results section. In addition HbA1c levels were broken down according to the risk categorisation proposed by the Irish College of General Practitioner Guidelines [9]. BMI results were categorised according to the WHO cut-off points [10].

Comparisons were drawn with the National Diabetes Audit of England for the corresponding period of 2008/2009. This is the largest annual audit of diabetes services in the world with over 1.5 million people with diabetes included, 75% of the diabetic population. All primary care trusts in England (N = 152) contributed data from 71% of GP practices (N = 5920) [11]. The NDA 2008/2009 data are contained on the NDA "Dashboard" where data are not broken down by type of diabetes. The published report contains some results stratified by type and where available figures for Type 2 diabetes are used. Data from the National Diabetes Audit of Wales were excluded from this study as results were based on data from 31% of practices in Wales and therefore were not considered representative.

Results were also compared to the Scottish Diabetes Survey, a population level survey published annually by the Scottish Diabetes Survey Monitoring Group. It collates nationally agreed data submitted by 14 NHS Boards in Scotland incorporating both primary and secondary care. Diabetes registers, held by each health board, are the main source of data for the survey. In 2009 over 220,000 people were included in the survey, of whom 87.4% had Type 2 diabetes. Results from 2009 were chosen as data were stratified by type of diabetes allowing for direct comparison with Type 2 diabetes management. Data on recording and outcomes were similar across the 2008 and 2009 Scottish Diabetes Survey [12].

### Statistical Analysis

Statistical analyses were undertaken using SPSS 16 (SPSS Inc., Chicago, IL, USA). Categorical data are presented as frequencies and percentage. Data are mean +/- SD for continuous variables. Between-group analysis of continuous variables was performed by an independent t-test. Categorical variables were analysed using the Pearson chi square test. A number of continuous variables were stratified into risk categories according to the national and international guidelines. There were missing data on a number of variables ranging from 6% non-recording for blood pressure to 36% for smoking status. Where this occurs, the figures represent the recorded data.

## Results

### Characteristics of the study participants

The profile of patients was similar to that reported in the National Diabetes Audit for England and the Scottish Diabetes Survey. Of the 3,010 patients, 56.5% were male (n = 1,701) (gender unknown for 0.4% of the sample, n = 11) comparable to 54.6% of males with diabetes in Scotland. Data on the gender breakdown of patients included in the NDA for England were not available.

The mean age of patients in this sample was 65.7 years (SD = 12.2). Over half of patients were aged 65 years or over (56.5%, n = 1691) compared to 70% of patients in Scotland. Twenty-five percent of the people included in the National Diabetes Audit for England were < 40 years old. There was a statistically significant age difference between males and females in this sample (64.7 vs. 67.1, p < 0.001).

### Recording Processes of Care

Process of care recording for clinical outcomes compared favourably to results from the National Diabetes Audit (NDA) for England and Wales, and the Scottish Diabetes Survey (Table 2). Recording for retinopathy screening (71%) was also similar to England and Wales (69%) and Scotland (80%). However recording was lower for body mass index (BMI) (70.4% vs. 90%) and foot screening (64.6% vs. 78.8% in Scotland). Only two of the three primary care initiatives collected information on the recording of smoking status. Among these practices (n = 1995), smoking status was recorded for 63% of patients compared to 99% recording in Scotland.

**Table 2:** Recording of process measures in practices compared to England, and Scotland.

	Three select primary care initiatives in Ireland		National Diabetes Audit England 2008/09	Scotland 2009 Type 2 DM %
	**% (n)**	**95% CI**	**%**	**%**

**HbA1c**	90.2*(2714)	89.0-91.2	91.7	89.9

**Blood Pressure**	93.5 (2814)	92.5-94.3	94.1	95

**Total Cholesterol**	92.2 (2776)	91.2-93.2	90.3	87.7

**LDL Cholesterol**	86.5 (2604)	85.2-87.7	-	-

**HDL Cholesterol**	84.9 (2554)	83.5-86.1	-	-

**Smoking Status (n = 1995)**	63.3 (1263)	61.5-65.4	86.8	99.3

**BMI**	70.4 (2119)	68.7-72.0	89.2	90.0

**Retinopathy Screening (n = 2629)**	71.2 (1872)	69.4-72.9	77.3	80.0

**Foot Assessment (n = 2292)**	64.6 (1481)	62.6-66.6	82.9	78.8

*4 patients with HbA1c = 0.1 were removed from analysis

†Data from the National Diabetes Audit dashboard represent Type 1 and Type 2 diabetes combined.

### Outcome measures

#### Glycaemic Control (HbA1c)

The mean HbA1c value for the sample was 7.1% (54 mmol/mol) (SD = 1.3) with no statistically significant difference in glycaemic control between males and females (p = 0.795). There was a significant relationship between the age of patients and glycaemic control (p < 0.01).

The mean HbA1c for patients with Type 2 diabetes across the 14 NHS Boards in Scotland was marginally higher (7.3% or 56 mmol/mol). Over one third of patients with Type 2 diabetes (35%, n = 943) reached the national recommended target for HbA1c (< 6.5% or < 48 mmol/mol), compared to 25% of patients with diabetes in England. Approximately 72% of patients had an HbA1c of < 7.5% (n = 1949) compared to 63.8% of patients with Type 2 diabetes in Scotland. Table 3 illustrates the stratification of patients into three risk categories for HbA1c levels.

**Table 3:** Patients categorized according to risk categories for HbA1c levels [9].

	Three select primary care initiatives in Ireland % (n = 2718)	National Diabetes Audit* England	Scotland** (%)
**Low Risk (< 6.5%)** **(< 48 mmol/mol)**	34.7 (943)	25.02	
			63.8 (< 7.5%)
**Medium Risk (6.5 - 7.5%)** **(48-59 mmol/mol)**	37.0 (1006)	37.8	

**High Risk (> 7.5%)** **(> 59 mmol/mol)**	28.3 (769)	37.2%	36.1

*National Diabetes Audit data represent Type 1 and Type 2 diabetes combined

**Scottish Diabetes Survey: HbA1c was categorised as < 7.5, (7.5-9.0) and > 9.0.

#### Blood Pressure

The mean systolic blood pressure for patients was 136.3 mmHg (SD = 16.6) with no significant difference between males and females (p = 0.786). Applying the cut-off of ≤ 140 mmHg for systolic blood pressure from the Scottish Diabetes Survey, 69% of patients in Ireland achieved this target compared to 74.6% of patients in Scotland. The mean diastolic blood pressure was 77.2 mmHg (SD = 9.3). Again there was no significant difference between males and females (p = 0.373). Seventy-two percent of patients (n = 1980) reached the national recommended target for diastolic blood pressure of ≤ 80 mmHg. Comparable data on diastolic blood pressure were not available from England or Scotland.

**Figure 1:**
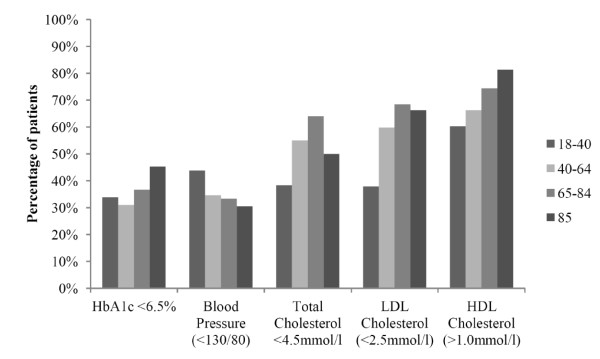
**Achievement of outcomes by age category**.

Thirty-seven percent of patients reached the recommended target for blood pressure in Ireland (≤ 130/80 mmHg). There was a significant association between achievement of the national blood pressure target and the age category of patients (p < 0.001) (Figure 1). More patients in the younger age category (18-40) reached the target of ≤ 130/80 mmHg compared to the other age groups: 18-40 (43.8%), 40-64 (34.6%), 65-84 (33.3%) and 85+ (30.5%). The target in the UK in 2008-2009 was ≤ 140/80 for patients without eye, kidney or vascular disease. Applying this target 54.4% of patients in this sample reached the target compared to 60.2% of patients with Type 2 diabetes in England.

#### Lipid Profile

The mean total cholesterol concentration for the group was 4.2 mmol/L (SD = 1.0). The mean LDL cholesterol concentration was 2.3 mmol/L (SD = 0.81) and the mean HDL cholesterol concentration was 1.2 mmol/L (SD = 0.36). Table 4 outlines the percentage of patients achieving national targets for total cholesterol, LDL cholesterol and HDL cholesterol. Using the Pearson chi square test, there were significant associations between gender and achievement of lipid targets (p < 0.001) and between patients' age category and the achievement of lipid targets (p < 0.001). Figure 1 illustrates the achievement of targets among the different age categories. Fifty-seven percent of patients from the primary care initiatives in Ireland achieved the UK total cholesterol target of < 4 mmol/l compared to 73.2% of patients with Type 2 diabetes in England

**Table 4:** Lipid profile of patients compared to national targets and comparing males and females.

	Recommended Target	Three select primary-Care Initiatives in Ireland %	Males vs. Females (%)	P value
**Blood Pressure**	≤ 130/80 mmHg	37%	33.6% vs. 34.5%	p = 0.35

**Total Cholesterol**	< 4.5 mmol/l	64.5%	69.8% vs. 56.6%	P < 0.001

**LDL Cholesterol**	< 2.5 mmol/l	64.2%	68.2% vs. 58.9%	P < 0.001

**HDL Cholesterol**	> 1.0 mmol/l	70.9%	62.6% v 82.0%	P < 0.001

#### Smoking

Smoking status was recorded in two of the three structured care initiatives (n = 1995) (Diabetes Interest Group, DIG and HSE Midland Area Diabetes Structured Care Programme). Within the two groups, smoking status was recorded for 63% of patients (n = 1263) compared to 87% recording in England and almost complete recording in Scotland (99.3%). Just over 1 in 5 people, who had their smoking status documented, were recorded as smokers (22.2%). There was no statistically significant difference between males and females (p = 0.364) (males = 22.9%, females = 20.8%). A similar smoking prevalence of 18.8% was reported in Scotland.

#### Body Mass Index (BMI)

**Figure 2:**
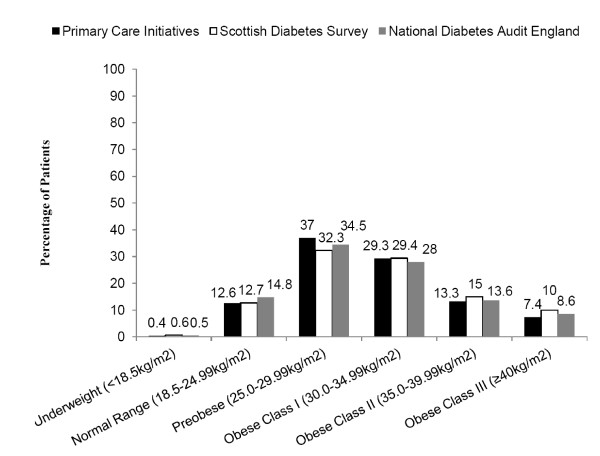
**Percentage of patients in each BMI category**.

The mean BMI of patients was 30.8 kg/m^2 ^(SD = 6.1), above the national target of < 25 kg/m^2 ^[7]. There was no statistically significant difference between males and females (30.6 kg/m^2 ^vs. 31.1 kg/m^2^, p = 0.082). Fifty-percent of patients with Type 2 diabetes (n = 1060) were in the obese category (> 30 kg/m^2^) compared to 54.4% of patients with Type 2 diabetes in Scotland (Figure 2).

## Discussion

### The quality of primary care-led diabetes management

This study has demonstrated what can be achieved by proactive and interested health professionals in the absence of a national infrastructure to support diabetes management. There was a high level of recording of intermediate clinical outcomes such as HbA1c and blood pressure, comparable to results in the UK. However the recording of lifestyle factors such as BMI and smoking status was markedly lower. Similar proportions of patients in Ireland and the UK were achieving targets for HbA1c, cholesterol and blood pressure. Over one third of patients reached the national target for glycaemic control (HbA1c < 6.5%) compared to one quarter of patients in the National Diabetes Audit for England (NDA). The proportion of patients meeting the UK blood pressure target (≤ 135/75 mmHg) was also similar to results in the National Diabetes Audit however it was less than 30% of patients in both instances. Half of all the patients were categorized as obese (> 30 kg/m^2^), similar to findings in Scotland.

The existence of the Quality Outcomes Framework (QOF) in the UK goes some way towards explaining the high level of process recording found in the National Diabetes Audit and the Scottish Diabetes Survey. However the proportion of patients in Ireland achieving targets for intermediate outcomes was similar to UK results, despite the lack of a comparable national incentive structure to improve the quality of care. This is in keeping with the suggestion that it is easier to improve the recording of care, which is under the control of the health professional, without necessarily making a significant impact on patient outcomes [13,14].

### The Role of Special Interest

The quality of care demonstrated by practices in this study highlights the contribution of special interest to the management of chronic diseases. The concept of special interest is a driver of quality assurance in the primary care initiatives as practice staff participate voluntarily in ongoing audit and feedback, a process hindered by the lack of stable and robust IT systems for routine data capture such as those available in Scotland [15]. The role of GPs with special interests has been formalized in the UK as part of the reconfiguration of services within the NHS to improve the accessibility, free up specialist hospital services and reduce waiting times [16,17]. It is also envisaged that GPs with special interests would take referrals from other GPs [18]. Evaluation of this policy direction is limited however the scheme has been piloted in the field of dermatology whereby a GP with a special interest achieved similar clinical outcomes to the hospital-based service, was more accessible and preferred by patients [19] although the cost of providing the specialist service in general practice was higher [20]. In Ireland formal specialisation is limited to nursing staff at present with calls for increasing numbers of Diabetes Nurse Specialists to facilitate the reorientation and reorganisation of diabetes care [21].

### Isolating the Improvement Factor

It is important to note that the essential ingredient for improving diabetes care has not been isolated. A recent review of systematic reviews of diabetes care programmes also failed to find conclusive evidence of the critical components of diabetes management programmes [22]. A number of studies have highlighted the positive impact of enhancing organisation and physician behaviour through multidimensional interventions [4,23]. Practices involved in these primary care initiatives have introduced a number of organizational and professional strategies, including patient registers and ongoing audit, which have been shown to be effective in improving delivery and outcome of care [4,24]. The search for the single × factor in quality improvement may be futile as strategies are rarely introduced in isolation and improvement may be a result of the synergy between different approaches [25]. Research has now begun to look towards what are the common features of high quality care. One common feature among the three initiatives involved in this study is the involvement of a nurse who is dedicated solely to supporting the delivery of evidence based diabetes care in the community.

### Strengths and Limitations

This study is limited in its comparisons by the dearth of information on the quality of diabetes care across Europe. It should be stressed that this study is not a comparison of 'like with like' but rather a benchmark of the performance of a select group of special interest practices providing structured care in Ireland against the standard of care observed in countries supported by a national diabetes management infrastructure. Furthermore the results of this study are not typical of diabetes care in Ireland. The lack of routine data collection in Ireland prohibited the inclusion of a reference group of practices not participating in initiatives delivering structured care.

This study focuses on the group-level performance of GPs involved in primary care diabetes initiatives however data on practice characteristics such as age and length in practice, which could influence the quality of care, were not available for analysis in this study. Future research should examine practice characteristics such as case load and staff levels which could discriminate between levels of performance within this group of interested GPs. The absence of an agreed core dataset for diabetes also limited the potential of the study as it was not possible to combine all data collected by the three initiatives. While data on long-term complications were available from two of the three groups, data collection has yet to be standardised and these outcomes are often not recorded consistently by all GPs involved. The overemphasis on intermediate outcomes measured in the short-term may underestimate the true effect of quality improvement interventions which have yet to be realised [26]. Data on long-term outcomes will contribute greatly to our understanding of the full extent of the impact of structured care and whether benefits have been sustained.

## Conclusions

This study highlights the quality of care that can be achieved by a group of proactive health professionals working together to provide evidence based care in the community in a system with minimal incentives. The recording of processes of care was similar to the UK with the exception of recording lifestyle factors, and similar proportions of patients achieved clinical targets. Primary care initiatives are a viable option for health systems trying to tackle the growing burden of diabetes care but we cannot presume or rely on special interest to improve the quality of care for all patients. The challenge facing health systems is to establish quality assurance as a responsibility for all health care professionals rather than the subject of special interest for a few.

## Competing interests

The authors declare that they have no competing interests.

## Authors' contributions

SMH, PM, KM, CC and CB conceived of the collaboration, participated in its design and coordinated the data collation. SMH played the lead role in reviewing the international literature, sourcing suitable comparators and drafting of the manuscript. KM, CC and CB played a major role in collecting and synthesizing the data in each audit region and PM led the analysis of the data and preparation of results. VH is a founding member of the HSE Midland Diabetes Structured Care Programme Midland Structured Care Programme and JM is a founding member of the Diabetes Interest Group. Both contributed a critical appraisal of the content. SMH and IP were responsible for drafting the manuscript while all authors were involved in the interpretation of findings and revision of the manuscript.

## Pre-publication history

The pre-publication history for this paper can be accessed here:

http://www.biomedcentral.com/1472-6963/11/348/prepub
